# Zika virus detection, isolation and genome sequencing through Culicidae sampling during the epidemic in Vitória, Espírito Santo, Brazil

**DOI:** 10.1186/s13071-019-3461-4

**Published:** 2019-05-08

**Authors:** Constância Flavia Junqueira Ayres, Duschinka Ribeiro Duarte Guedes, Marcelo Henrique Santos Paiva, Mariana Carolina Morais-Sobral, Larissa Krokovsky, Laís Ceschini Machado, Maria Alice Varjal Melo-Santos, Mônica Crespo, Cláudia Maria Fontes Oliveira, Ricardo Silva Ribeiro, Orlei Amaral Cardoso, Ana Lúcia Barbosa Menezes, Roberto Costa Laperrière-Jr, Carlos Feitosa Luna, André Luiz Sá Oliveira, Walter Soares Leal, Gabriel Luz Wallau

**Affiliations:** 1 Departamento de Entomologia, Instituto Aggeu Magalhães (IAM), Fundação Oswaldo Cruz, Recife, Brasil; 2 Universidade Federal de Pernambuco, Caruaru, Brasil; 3 Núcleo de Vigilância em Saúde, Superintendência Regional de Saúde de Vitória, Cariacica, Brasil; 4 Centro de Vigilância em Saúde Ambiental, Secretaria Municipal de Saúde, Vitória, Brasil; 5 Núcleo Especial de Vigilância Ambiental, Gerência Estratégica de Vigilância em Saúde, Secretaria de Estado da Saúde, Vitória, Brasil; 6 Núcleo de Estatística e Geoprocessamento (NEG), Instituto Aggeu Magalhães (IAM), Fundação Oswaldo Cruz, Recife, Brazil; 7 Department of Molecular and Cellular Biology, University of California, Davis, USA

**Keywords:** Field-caught mosquitoes, Zika virus, RT-qPCR, Genome sequencing

## Abstract

**Background:**

Zika virus (ZIKV) has been isolated from many mosquito species in nature, but it is believed that the main vectors in urban environments are species of the genus *Aedes*. Here, we detected and isolated ZIKV in samples from *Aedes aegypti*, *Aedes taeniorhynchus* and *Culex quinquefasciatus*, collected during the Zika epidemic in Vitória, southeast Brazil. Using quantitative real-time polymerase chain reaction, ZIKV detection was performed in mosquito samples collected from February to April 2016.

**Results:**

Overall, six pools of mosquitoes were positive for ZIKV: four of *Cx*. *quinquefasciatus*, one of *Ae. aegypti* and one of *Ae. taeniorhynchus*. Their genomes were sequenced.

**Conclusions:**

These results support and strengthen the hypothesis that other mosquito species can also be involved in ZIKV transmission.

## Background

In 2015–2016, the world faced the spread of a global epidemic caused by Zika virus (ZIKV). This arbovirus (arthropod-borne virus) was first identified in Uganda in 1947 from sentinel monkeys but remained unnoticed in humans until 60 years later [1]. In 2007, a Zika outbreak was reported in Micronesia, affecting 73% of the inhabitants, followed by a new outbreak in French Polynesia in 2013, which affected 70% of the population [2, 3]. Despite these reports, no severe consequences of ZIKV infections in humans had been described until its arrival in South America. In this continent, ZIKV infections were associated with birth defects, due to mother-to-child transmission of the virus, such as microcephaly and other neurological disorders, which are now collectively referred to as “congenital Zika syndrome” [4, 5]. In addition, ZIKV is also capable of triggering neurological abnormalities, such as Guillain–Barré syndrome [6]. From a mild disease before 2015 to an epidemic that has a diverse impact on population health, in 2016 the World Health Organization (WHO) declared Zika a public health emergency of international concern [7].

The ZIKV is a positive-sense, single-stranded RNA virus, with a genome length of approximately 11 kb, which codes for a single polyprotein [3] composed of three structural proteins: capsid (C), pre-membrane (prM) and envelope (E); as well as seven nonstructural proteins: NS1, NS2a, NS2b, NS3, NS4a, NS4b and NS5 [8]. The structural proteins are responsible for the formation of the virus particle, while non-structural proteins are responsible for important functions in genome replication, polyprotein processing and host response [9]. ZIKV transmission primarily occurs through bites from infected mosquitoes, but unlike other arboviral diseases, other non-vector-borne transmission pathways have been reported, such as blood transfusion, sexual transmission, transplacental infection and breastfeeding [10–13]. In addition, studies have demonstrated the presence and persistence of infective ZIKV in saliva, urine and semen [8, 14].

*Aedes aegypti* is considered the main vector of ZIKV along with *Aedes albopictus* [15–18]. However, *Culex quinquefasciatus*, the most common mosquito in urban areas of the southern hemisphere, also known as the southern house mosquito, was implicated in ZIKV transmission during recent epidemics [19]. This mosquito’s vector competence has been recently demonstrated under laboratory conditions by three different groups [20–22], as well as in field samples, when ZIKV was isolated from two pools of *Cx. quinquefasciatus* mosquitoes from Recife (northeast region), and from several pools collected in Guadalajara, Mexico [23].

Here, we report the results of a study conducted in early 2016 to detect ZIKV in field-caught mosquitoes collected in the city of Vitória, Espírito Santo, Brazil.

## Results

**Fig. 1:**
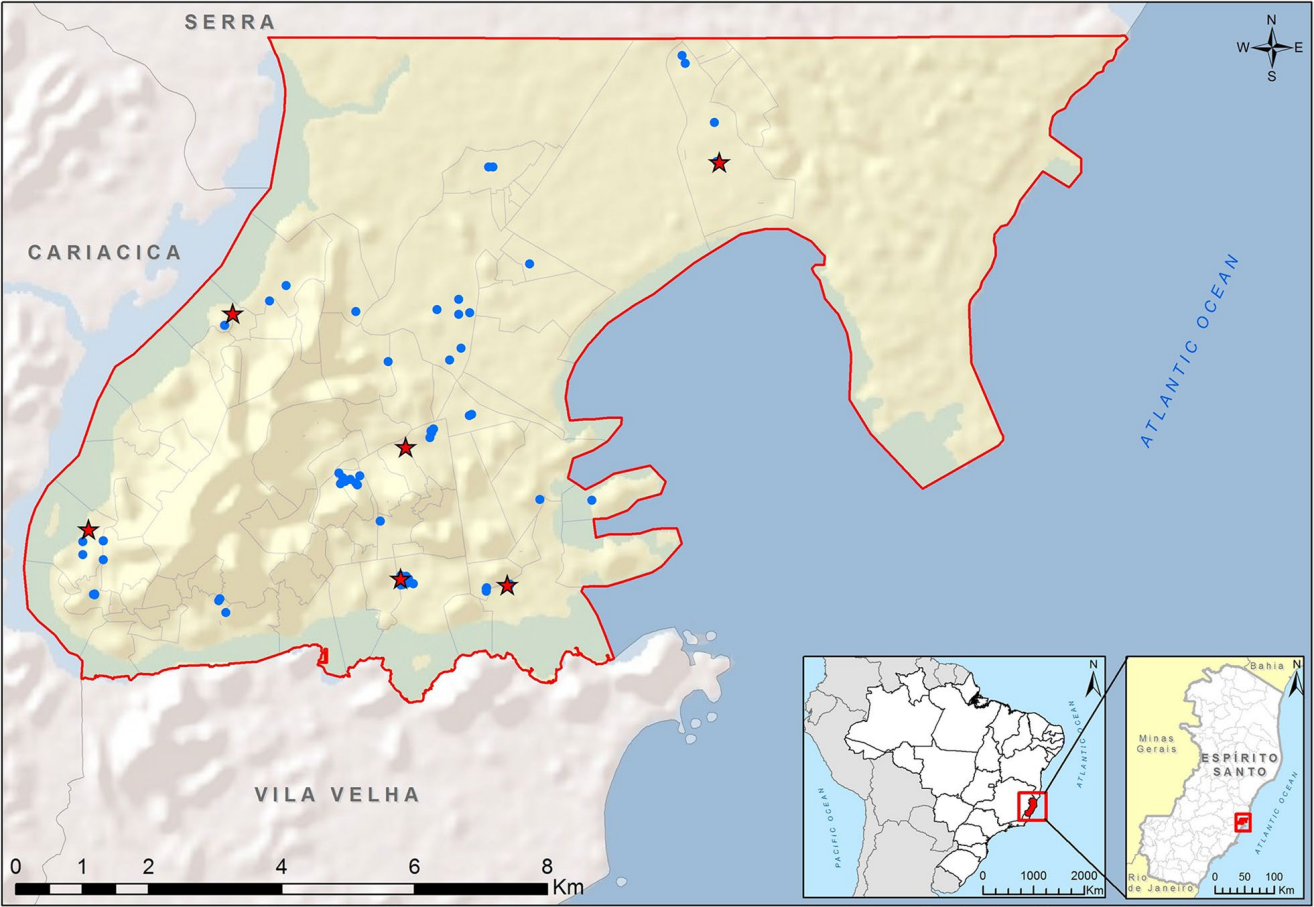
Location of the city of Vitória, capital of Espírito Santo State, indicating both mosquito collection sites (blue dots) and the places where ZIKV-positive samples were detected (red stars). The map was produced using ArcGIS v.10.6 (Esri, Redlands, CA, USA)

Aspirations resulted in the highest number of collected mosquitoes among the three collection methods: *Cx. quinquefasciatus* (*n* = 223), *Ae. aegypti* (*n* = 12), *Aedes taeniorhynchus* (*n* = 6) and *Aedes scapularis* (*n* = 1). *Culex quinquefasciatus* was also the most abundant mosquito collected in CDC traps (*n* = 47), followed by *Ae. taeniorhynchus* (*n* = 19). No other *Aedes* species were collected in CDC traps. New Jersey traps collected more *Ae. taeniorhynchus* (*n* = 20) than *Cx. quinquefasciatus* (*n* = 16) and *Ae. aegypti* (*n* = 2). Figure 1 shows the distribution of sites where mosquitoes were captured and positive pools were detected.

A total of 346 mosquitoes were analyzed in 95 pools, of which 25 (26%) contained mosquito females that had recently taken a blood meal, and 70 (74%) contained mosquito females without visible blood remains in the abdomen. Six pools from the second group (non-fed mosquitoes) were positive for ZIKV (Table 1).

**Table 1:** Zika virus infection of potential vectors collected in Vitória-ES

Mosquito species	No. of pools	No. of females (no. of non-fed females)	Positive pools (Ct values)	Sampling method	MIR^a^
*Ae. aegypti*	8	14 (5)	1 (37.1)	Aspiration	200
*Ae. taeniorhynchus*	27	45 (1)	1 (37.0)	CDC	22.72
*Ae. scapularis*	1	1 (0)	0	–	–
*Cx. quinquefasciatus*	59	286 (102)	4 (37.3; 37.2; 37; 37.9)	CDC (1), NJ (1), aspiration (2)	39.2
Total	95	346	6		–

^a^MIR was calculated using pools that contained only non-blood-fed females

**Fig. 2:**
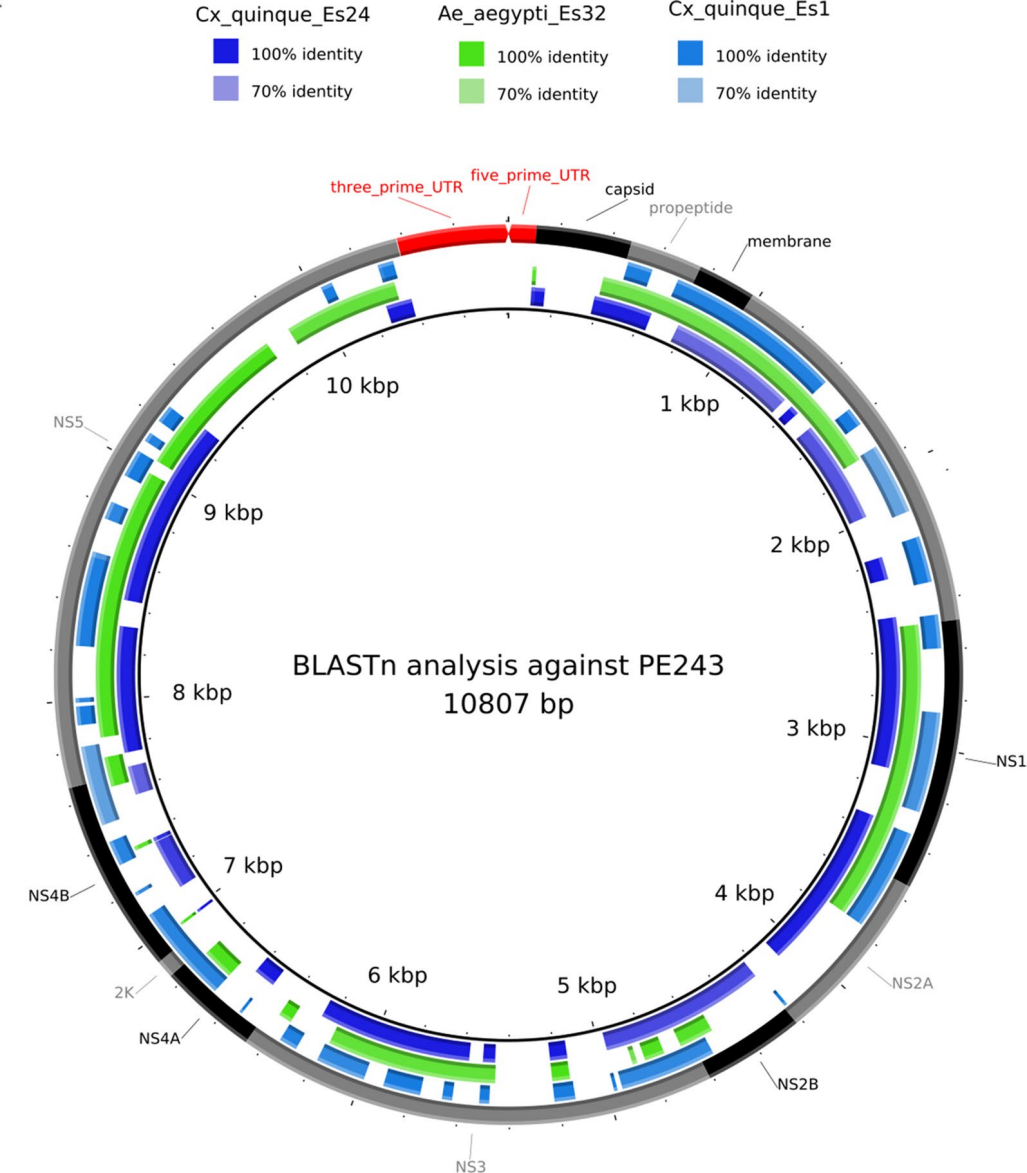
Circular representation of the ZIKV genome (PE243: KX197192) and its corresponding annotation (black and gray external rings, red arrow denotes the UTRs of ZIKV) along with blastN (default parameters) of partial genomes obtained from *Cx. quinquefasciatus* (bluish rectangles) and *Ae. aegypti* (greenish rectangles)

ZIKV was isolated after the first passage in Vero cells from three out of the six positive pools collected in Vitória (two were obtained from *Cx. quinquefasciatus* and one from *Ae. aegypti*). None of these pools showed signs of recent blood-feeding. These genomes were named ZIKV/*C. quinquefasciatus*/Brazil/ES01/2016 (Cxq_ES1), ZIKV/*C. quinquefasciatus*/Brazil/ES24/2016 (Cxq_ES24) and ZIKV/*Ae. aegypti*/Brazil/ES32/2016 (Aea_ES32). Sequencing efforts obtained partial ZIKV genomes, which were aligned against all ZIKV genomes available from Faria et al. [24]. ZIKV partial genomes are available in the figshare website, as NGS reads (https://figshare.com/s/965be7f2e89dafa8885a). These genomes presented a 44% (4745) coverage breath for Cxq_ES1, 57% (6157) for Cxq_ES24 and 55% (5948) for Aea_ES32 (Fig. 2). The coverage depth obtained ranged from 19× for Aea_ES32 to 1029x for Cxq_ES1, with Cxq_ES24 sequenced at an intermediate depth of approximately 222×.

**Fig. 3:**
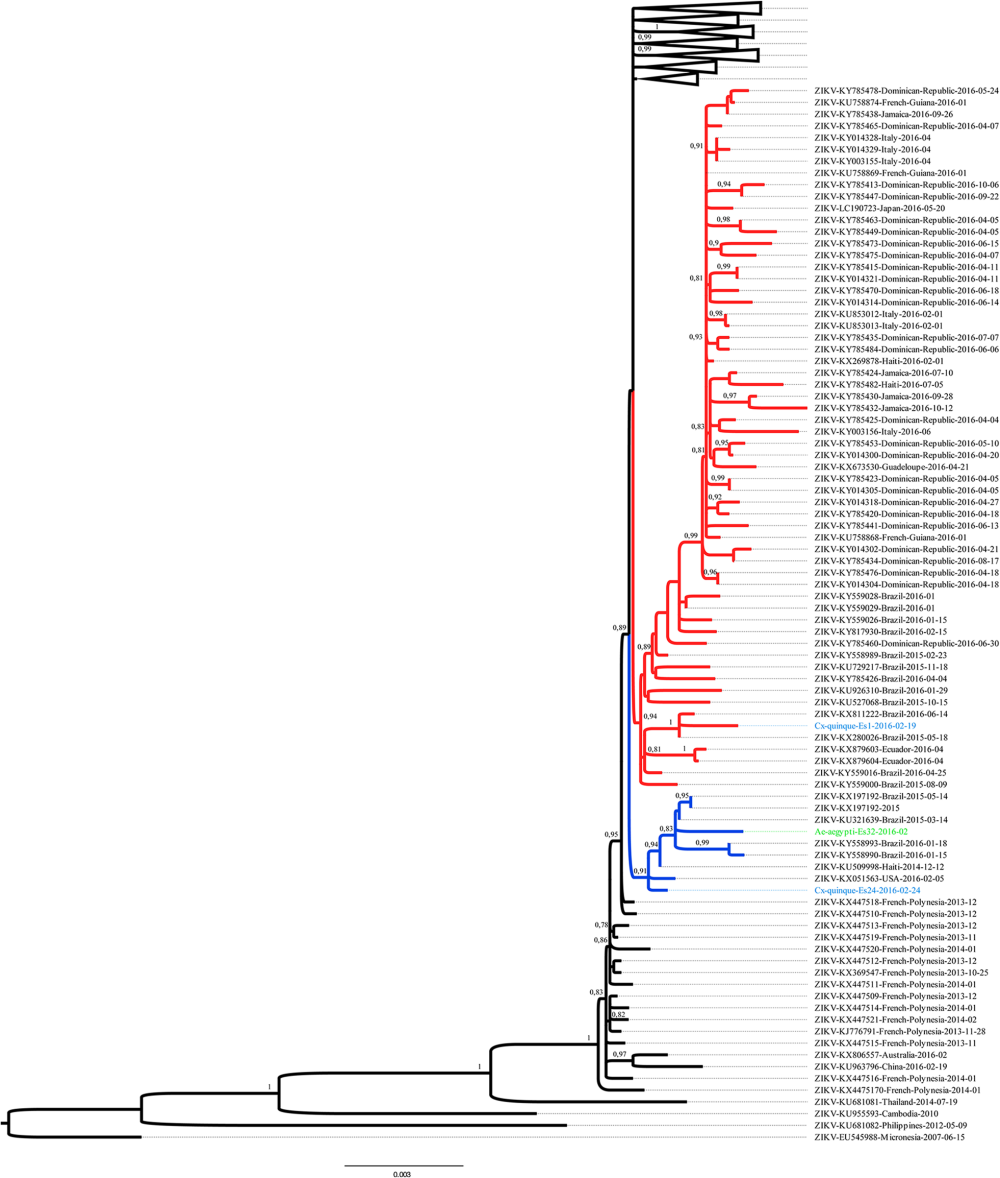
ZIKV maximum likelihood phylogenetic reconstruction of the Asian genotype: blue branches represent an early offset of ZIKV in Brazil where the CXq_ES24 and Aea_ES32 genomes clustered and red branches denote one of the clades of ZIKV which emerged during the 2015 epidemic where CXq_ES1 clustered. SH-like branch support is depicted above or in front of the corresponding nodes and only aLRT branch support above 0.80 are shown

Phylogenetic analysis of all available ZIKV genomes revealed that two sequenced ZIKV genomes (Cxq_ES24 and Aea_ES32) clustered with an early clade of ZIKV detected in Haiti in 2014 and in Pernambuco state in 2015 (Fig. 3) [full maximum likelihood tree can be seen on the Interactive Tree of Life website (https://itol.embl.de/tree/19118716467351551538327490)]. In addition, the Cxq_ES1 ZIKV genome clustered within one of the major ZIKV branches that caused the 2015 epidemic in Brazil (Fig. 3). Of note, Cxq_ES24 draft genome differed by seven single nucleotide polymorphisms (SNPs) from the reference PE243 genome [25], while Aea_ES32 differed by 12 SNPs from the PE243 ZIKV genome. Because Cxq_ES1 clustered together with strains of another clade, we compared it with the Paraíba ZIKV genome (KX280026) and observed the presence of seven SNPs. Considering all complete and draft genomes of ZIKV used in the alignments, the sequences obtained in this study showed non-exclusive SNPs, that is, such variants also occurred in most ZIKV genomes from the 2015–2016 epidemic (data not shown).

## Discussion

In this study, we report the detection of ZIKV in field-caught mosquitoes from Vitória, (ES), during part of the 2016 epidemic. ZIKV was found in three different species, namely *Ae. aegypti*, *Ae. taeniorhynchus* and *Cx. quinquefasciatus*. This is the first report of ZIKV detection in species other than *Ae. aegypti* in southeast Brazil.

The identification of ZIKV mosquito vectors has been the subject of a heated debate. The proof-of-principle study performed by Guo et al. [21] demonstrated that transmission by *Cx. quinquefasciatus* is feasible, but only evidence collected in natural settings, such as during outbreaks, can demonstrate how this species contributes to ZIKV spread. Unfortunately, despite the very high incidence of Zika in human populations, there is a lack of data regarding the spatial and temporal association of human cases and local vector abundance, preventing us from concluding which mosquito species plays the dominant role in ZIKV transmission in this urban context. From the 81 countries reporting recent ZIKV outbreaks, it was only possible in three (Brazil, Mexico and USA) to detect ZIKV circulating in vector populations. In Brazil, ZIKV has been detected in *Ae. aegypti* from Rio de Janeiro [26] and in *Ae. aegypti* and *Cx. quinquefasciatus* from Recife [20]. In Mexico, 15 out of 58 pools of *Ae. aegypti* were found positive for ZIKV in Chiapas state [27]. In Guadalajara, Mexico, ZIKV has been isolated from several mosquito species, especially *Cx. quinquefasciatus* [23]. Before the recent ZIKV outbreaks, *Culex perfuscus* was found naturally infected with ZIKV in Senegal and this species displayed a MIR tenfold higher than *Ae. aegypti* [28].

The results obtained here give further support for the role of both *Ae. aegypti* and *Cx. quinquefasciatus* in the ZIKV transmission. Our group also reported in a previous paper that *Cx. quinquefasciatus* plays an important role in ZIKV spread in Brazil through laboratory and field experiments [20]. On the other hand, several research groups have conducted vector competence studies of *Culex* and *Aedes* mosquitoes and demonstrated that *Cule*x species are not competent to transmit ZIKV [29–34]. The nature of these conflicting results should be investigated in more detail, as different explanations have been suggested [35, 36]. A study conducted in Florida, USA, to identify the pattern of host use by mosquito species, suggested that *Cx. quinquefasciatus* plays a secondary role in ZIKV transmission, and that *Ae. aegypti* and *Ae. albopictus* are the most likely ZIKV vectors. The authors concluded that the relative importance of each species in spreading the virus may vary according to specific location and population [37].

Considering that *Cx. quinquefasciatus* may play a role in ZIKV transmission, it is crucial to highlight the distinct biological traits displayed by *Culex* and *Aedes* mosquitoes. The population distribution of these mosquitoes differs in most Brazilian cities and most tropical countries; *Cx. quinquefasciatus* is approximately 20 times more abundant than *Aedes* as shown previously [38, 39]. In addition, *Cx. quinquefasciatus* females show different feeding behavior when compared to *Ae. aegypti* females. Usually, an *Ae. aegypti* female can feed on multiple hosts until its gonotrophic cycle is complete [40], while *Cx. quinquefasciatus* usually feed on only one host [41]. This behavior increases the potential for *Ae. aegypti* to become infected compared to *Cx. quinquefasciatus*.

On the other hand, *Cx. quinquefasciatus* mosquitoes in the southern hemisphere exhibit a highly anthropophilic feeding behaviour. In Brazil, this species is solely responsible for the active transmission of *Wuchereria bancrofti*, the lymphatic filariasis causative agent [42]. It has also been implicated as a potential vector in the transmission of Oropouche virus (OROV), in the state of Mato Grosso [43], which was considered the second most common arbovirus in Brazil [43, 44].

In this study, we detected a pool of *Ae. taeniorhynchus* positive for ZIKV, but we were unable to isolate and sequence the ZIKV from this pool. *Aedes taeniorhynchus* is a common species found along the Brazilian coastline, and it is implicated as the primary vector of canine heartworm disease in the northeastern and southeastern regions of the country [45]. In addition, it has been identified as the primary vector of Venezuelan equine encephalitis [46] and as a potential vector for maintaining the transmission cycle of West Nile fever (WNV) in the Galapagos [47]. Further studies are necessary to better understand if *Ae. taeniorhynchus* play a role in ZIKV transmission among human.

In order to obtain detailed information about the viral strains circulating in this region we performed isolation of ZIKV from field-caught mosquitoes and sequenced their genome. Bioinformatics analysis showed that these strains were clustered into two distinct clades. The first comprised Cxq_ES24 and Aea_ES32 along with the virus Brazil PE243 2015 (from Pernambuco state), Haiti 1225 2014 and SPH2015 (from São Paulo state). This clade encompasses the first group of ZIKV strains identified in northeast Brazil. The second clade clustered Cxq_ES1 with several other strains reported in the southeast and northeast Brazil. Interestingly, the detection of strains from the first clade in other Brazilian state, besides Pernambuco and São Paulo, shows that those variants also are circulating and spreading very rapidly in comparison to the other epidemic clades [24].

Previous data suggest that ZIKV is a very fast-evolving and adaptive virus, and we should not expect it to fit into previously described simple models (i.e. one pathogen-one vector). Instead, the scientific community should keep an open mind [48] and focus on understanding the ecology of ZIKV and its interactions with different host cells (i.e. human, non-human vertebrates and mosquitoes) to elucidate its amplification cycles in urban environments and to prevent new ZIKV outbreaks and its associated neurological diseases.

## Conclusions

Results found in this study confirm the detection of ZIKV in field-caught mosquitoes from Vitória, Espírito Santo (ES) in three different mosquito species (*Ae. aegypti*, *Ae. taeniorhynchus* and *Cx. quinquefascitus*). Additionally, this is the first report of ZIKV detection in species other than *Ae. aegypti* in the studied area. Furthermore, it was observed that ZIKV strains were clustered into two distinct clades, showing that these variants are co-circulating and spreading very rapidly. Thus, it is of utmost importance to detect and monitor the emergence and spread of ZIKV in the country to implement successful surveillance strategies.

## Methods

### Study site

Vitória is the capital of Espírito Santo, a state located in the southeastern region of Brazil (Fig. 3). This municipality is a river-oceanic archipelago of 63,396 km^2^, consisting of 35 islands and a mainland region with an estimated population of almost 360,000 inhabitants [23, 49]. Approximately 40% of its area is covered by hills. Vitória comprises 80 neighborhoods, grouped into nine administrative regions. The city is considered the second best Brazilian capital with respect to quality of life indicators. The climate in Vitória is tropical with two distinguishable periods: the wet season from October to December and the sub-dry season from January to September. The exception is August, which is the driest month of the year.

### Mosquito collections

Adult female mosquitoes were collected from February to April 2016 using three different methods: New Jersey and CDC light traps and battery-powered aspirators. Thirty-seven CDC light traps, with white lights, were installed for surveillance and control of *Culex* spp. in 35 neighborhoods in Vitória. In addition, nine New Jersey light traps were installed in other districts that had reported high numbers of Zika cases. All traps were installed outdoors at public building properties, to facilitate access for the community health agents at the time of sample collection. Traps were turned on late in the afternoon and disconnected early the next morning after sample collection.

Battery-powered aspirators were also employed during daytime for capturing adult female mosquitoes in schools, hospitals, basic healthcare units and residences, to block Zika transmission in areas where Zika cases had been reported. Mosquitoes caught in New Jersey and CDC light traps were collected by vector control staff members, while municipal health agents involved in combating endemic diseases performed aspirations. Samples were identified from the traps and transported to the laboratory immediately after collection. After being anaesthetized at −20 °C for 10 min, mosquitoes were placed on Petri dishes on ice and sorted by species, sex, date and location. Females were pooled into 1.5 ml microtubes with a maximum of 20 females per tube. Then, 100 µl of RNAlater^TM^ stabilization solution was added to each sample, which were then covered with cotton balls and stored at −20 °C. In September 2016, samples were shipped to Aggeu Magalhães Institute (FIOCRUZ-PE) in Recife, in portable thermal iceboxes filled with dry ice.

### RNA extraction and RT-qPCR

After registering feeding status, mosquitoes were re-pooled into groups of a maximum of 10 specimens. RNA extractions and ZIKV molecular detection (RT-qPCR description and protocol) are described in Guedes et al. [20] and in Lanciotti et al. [3]. All samples were tested in duplicate. A reference sample (ZIKV BRPE243/2015 RNA) was used as a positive control, and a negative control consisted of RNA from a known ZIKV-negative mosquito pool. Both positive and negative controls were used in all RT-qPCR assays.

### Minimum infection rate (MIR)

To estimate viral infection rates in mosquito samples, we calculated the minimum infection rate (MIR), which is the number of positive pools divided by the total number of specimens tested and multiplied by 1000 as previously described [50].

### Spatial analysis

Collection sites were georeferenced based on an address geocoding method that transforms residential addresses registered in a tabular database into geographic coordinates, by first using a database of streets stored in a digital cartographic base and then converting them to geographical coordinates (latitude and longitude) using QGIS, a free geographical information system (GIS) software. Therefore, georeferenced information was derived by registering each address displaying an associated geographical coordinate for each location. ArcGIS v.10.6 was used to create a map.

### Isolation and sequencing of ZIKV from field-collected samples

ZIKV-positive pools from non-fed mosquitoes were used to recover ZIKV strains according to the protocol described in Guedes et al. [20]. To obtain ZIKV genomic sequences from positive samples, we performed the conditions described in the ZIBRA project protocol V3 [51]. PCR products were quantified using a Qubit dsDNA HS Assay Kit (Thermo Fisher Scientific Inc., Waltham, USA). MiSeq (Illumina, San Diego, CA, USA) sequencing libraries were prepared with a Nextera XT Library Prep Kit (Illumina) using 2 ng of input cDNA derived from the ZIKV multiplex PCR, following manufacturer’s instructions. MiSeq Reagent Kit V3 (Illumina; 150 cycles) was used in a paired-end strategy, resulting in 75 bp reads separated from each other by ca. 350 bp. Sequencing was performed in a MiSeq instrument (Illumina) from the Technological Platform Core at Aggeu Magalhães Institute.

### ZIKV genome analysis

Low quality raw reads, adaptors and ZIKV primers were trimmed with Trimmomatic v 0.36 [52], using the ILLUMINACLIP parameter. Bowtie2 [53] was used to map reads against the reference genome PE243 (KX197192.1) and consensus sequences were generated with Integrated Genome Viewer (IGV) [54, 55], considering only regions with coverage depth higher than 5x.

### Phylogenetic analysis

Phylogenetic reconstruction was performed with PhyML 3.0 [56] using the GTR + I + G nucleotide substitution models suggested by the SMART model selection [57]. The tree topology search was performed with NNIs and SPRs [58]. Branch support was evaluated with the aLRT [59]. A total of 255 draft and complete available ZIKV genomes, were collected from NCBI (https://www.ncbi.nlm.nih.gov/) up to December 2017, aligned with Mafft online service [60] and manually edited to keep only the ZIKV coding region. Figure generation was performed with FigTree (http://tree.bio.ed.ac.uk/software/figtree/).

## Abbreviations

ZIKV: Zika virus
RT-qPCR: quantitative real-time polymerase chain reaction
WHO: World Health Organization
CDC: Centers for Disease Control and Prevention
Ct: threshold cycle
MIR: minimum infection rate
SNP: single nucleotide polymorphisms
OROV: Oropouche virus
WNV: West Nile virus
